# Reduced graphene oxide paper electrode for lithium-ion cells – towards optimized thermal reduction

**DOI:** 10.3762/bjnano.17.3

**Published:** 2026-01-05

**Authors:** Agata Pawłowska, Magdalena Baran, Stefan Marynowicz, Aleksandra Izabela Banasiak, Adrian Racki, Adrian Chlanda, Tymoteusz Ciuk, Marta Wolczko, Andrzej Budziak

**Affiliations:** 1 Flake Graphene Research Group, Łukasiewicz Research Network – Institute of Microelectronics and Photonics, al. Lotników 32/46, 02-668, Warsaw, Poland; 2 AGH University of Krakow, al. Adama Mickiewicza 30, 30-059, Krakow, Poland; 3 Warsaw University of Technology, Faculty of Chemical and Process Engineering, ul. Waryńskiego 1, 00-645, Warsaw, Poland; 4 Warsaw University of Technology, Faculty of Chemistry, ul. Noakowskiego 3, 00-664, Warsaw, Poland; 5 SiC Technologies Research Group, Łukasiewicz Research Network – Institute of Microelectronics and Photonics, al. Lotników 32/46, 02-668, Warsaw, Poland

**Keywords:** electrode material, graphene paper, lithium-ion batteries, reduced graphene oxide, thermal reduction

## Abstract

This work introduces the results of characterizing free-standing reduced graphene oxide paper, given its potential use as an electrode material in lithium-ion cells. Mildly reduced graphene oxide paper underwent further thermal reduction steps. The structural and chemical properties of the obtained materials were determined using Raman and Fourier-transform infrared spectroscopies and elemental combustion analysis. The morphology and thickness were determined with scanning electron microscopy imaging. This paper also reveals electrical and electrochemical properties of the material. The conductivity of the material obtained at 800 °C reached ≈70 S/cm, and the discharge capacity reached ≈160 mAh/g at 100 mA/g current density.

## Introduction

Electrode materials comprising reduced graphene oxide (rGO) for energy storage in lithium-ion-based or sodium-ion-based technologies have been the subject of over 3800 publications between 2020 and 2024 in the ScienceDirect database only [1]. Market forecasts predict the growth of the overall rGO market at a compound annual growth rate of 36.5% between 2024 and 2031 [2].

**Figure 1:**
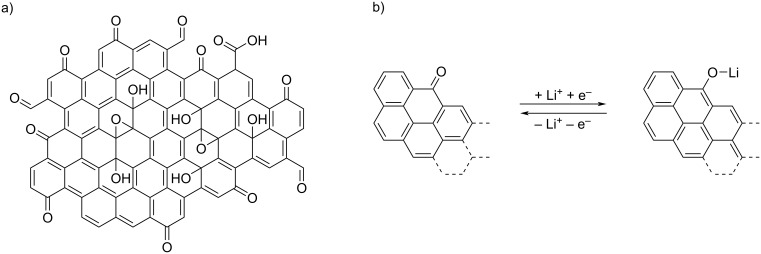
(a) Scheme of a reduced graphene oxide flake structure with examples of oxygen functionalities, that is, ketone (C=O), epoxide (C–O–C), carboxyl (COOH), hydroxy (OH), and aldehyde (CHO) groups. (b) Suggested redox reaction scheme [6,24–25]. This scheme was prepared with the help of the ChemDraw Ultra tool [26].

This material owes its popularity in particular to such properties as sufficient electrical conductivity and specific surface area, and low bulk density [3–4]. The role of rGO in electrode materials can be considered in two ways. It can be applied as a conductive additive, improving transport properties and, as a result, enhancing capacity [5]. It can also be the active material itself [6–7]. To mention just a few examples of rGO-based materials for electrodes, especially for lithium-ion batteries, LiFePO_4_ and rGO composite cathodes were reported by Wi et al. and Wei and colleagues [8–9]. Moreover, Wang et al. described a method for LiMn_0.75_Fe_0.25_PO_4_ nanorod production on rGO sheets [10]. Again, the carbonaceous base material was chosen due to its high conductivity. The relevance of rGO is not limited to only one electrode; Kiran et al. revealed performance enhancement of both cathode and anode materials for hybrid supercapacitors as a result of rGO application as core structure [11]. A similar approach, that is, rGO as conductive support, was presented by Thangappan et al. for nanostructured MoS_2_ in supercapacitor electrodes [12]. Another application of graphene materials is current collectors for both anode and cathode based on CVD-grown graphene foam, as described by Li et al., who reported lithium-ion batteries with Li_4_T_5_O_12_ and LiFePO_4_ active materials for the electrodes [13]. Chen et al. described current collectors based on rGO films [14]. Reduced graphene oxide finds further use in sodium-ion composite cathode materials as a conduction-improving agent with Prussian white as the active material [15]. Furthermore, advanced materials for dry electrode materials in all-solid-state batteries were reported, with the graphene additive serving as conductivity-improving agent [16–18]. Reduced graphene oxide was also investigated by Ma et al. as a modification of the separator materials in lithium sulfur batteries [19]. Such an improvement is enabled by the graphene-like structure and the defects within the flakes, represented not only by non-regular rings in the carbon lattice but also by the presence of oxygen functionalities and their percentile content [20–22]. A schematic illustration of these functional epoxide, ketone, hydroxy, and carboxyl groups on the surface of a rGO flake is shown in Figure 1a. In addition, the adjustability of lateral size and thickness of the rGO flakes, which influence functional parameters [23], is another advantage of these materials.

According to the literature, it is the ketone group that is characterized by reactivity and participates in redox reactions [6,24–25]. The suggested redox reaction scheme is depicted in Figure 1b. During discharge, lithium ions are transported through the electrolyte to the cathode material, where they react with the oxygen in the ketone groups and the electrons from the external circuit, creating Li-terminated chains. From a practical point of view, it is crucial to ensure an increased number of ketone groups by optimizing the reduction process of graphene oxide. Acik et al. presented a detailed description of oxygen species and their behavior during the thermal annealing of GO, allowing them to specify a temperature range expected to provide optimal type and amount of oxygen species [27]. According to this article, the desired ketone groups partially remain, while carboxyl, epoxy, and hydroxy groups are removed after thermal reduction at 850 °C. The research presented herein extends the scope of the cited article since it concerns the structural, chemical, and physical properties of free-standing rGO thin films (i.e., reduced graphene oxide paper) with the practical purpose of performance parameter testing. Thermal reduction of rGO paper sheets poses a challenge in preventing the material from burning and losing its continuity.

Removal of functional groups can be achieved by various methods, including chemical, thermal, electrochemical, or microwave processing [28]. Both chemical and eletrochemical reduction methods may result in a more defective structure of the graphene material [29–30], which, in this case, should be considered as a shortcoming of such an approach. Thermal methods may be additionally beneficial as they promote changes in the material's morphology introducing air pockets [31] and flake edge exposition on the rGO paper surface, improving accessibility of the redox sites for electrolyte and lithium ions. Microwave methods, which also lead to changes in morphology (i.e., expansion) of the material, require thermal pre-treatment since GO weakly absorbs incident microwaves [32]. This makes the production process longer and more expensive.

In this work, we provide an insight into the chemical, structural, electric, and electrochemical properties of rGO paper influenced by various thermal reduction processes. The presented results contribute to optimizing the technology of rGO paper regarding its application as an electrode material in lithium-ion batteries. Considering the possible implementation of this material as an electrode in secondary cells, its additional advantages in terms of the production process are also worth mentioning. The applied method of preparation [33] is scalable and does not require any toxic solvents, binding agents or chemicals for the reduction process. Moreover, one expects the functionalization to improve the stability and capacity of the final material.

## Experimental

### Graphene oxide paper preparation

To begin with, the G-Flake^®^ graphene oxide paper was produced in the Flake Graphene Research Group in Łukasiewicz Research Network, Institute of Microelectronics and Photonics, Warsaw, Poland, according to a patented method [33], based solely on graphene oxide paste without any plasticizer. This method of thin film preparation is easy to scale up and, which is worth emphasizing, eliminates the use of any binder or harmful solvent. In order to obtain rGO paper sheets, the produced paper sheets underwent a mild, three-step thermal procedure described in [34] (i.e., sample M300 mentioned in the current article) with further modification, that is, an additional thermal step was applied. For this research, three different additional steps were applied, namely, 400, 600, and 800 °C (the samples were named accordingly T400, T600, and T800). The additional thermal step was conducted for 6 h in an inert atmosphere in a tube furnace.

### SEM imaging

The morphological properties of the rGO paper were described based on SEM imaging (Phenom ProX). The thicknesses of the obtained sheets were determined from SEM images of the samples’ cross sections as an averaged value calculated over 50 length readings.

### TGA

TGA Q5000 equipment was applied for thermogravimetric analysis. The measurement was performed in an inert gas flow. The heating rate in this experiment was set to 3 °C/min with a final temperature of 800 °C. The sample was cut to fit the platinum pan.

### Elemental combustion analysis

A LECO O836 analyzer was utilized to determine the oxygen mass percentage in each G-Flake^®^ rGO paper sample. The equipment was calibrated with standard silicon oxide samples before each measurement. The samples of the measured materials were prepared to follow the requirement of the sample’s weight exceeding 0.01 g. The experiment involved four samples per material, that is, M300, T400, T600, and T800.

### Raman spectroscopy

The structural properties of the rGO paper sheets were studied with Raman spectroscopy (InVia Renishaw spectrometer). The spectra were collected with three accumulations, a 10 s exposure time, and 1% laser power (laser wavelength: 532 nm) in the 100–3200 cm^−1^ wavenumber range.

The data analysis involved normalization (0 to 1) of the measured spectra and a peak fitting procedure. The sum of Gaussian–Lorentzian functions (referred to also as pseudo-Voigt functions applied for the A, D, D'', B, 2D, D+G, and C peaks) and a Pearson type-IV function (applied for the G peak due to its asymmetry) was fitted [21,35–36].

The applied Gaussian–Lorenztian function


[1]

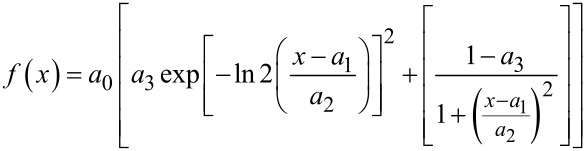



required the fitting of the following parameters: the peak’s amplitude *a*_0_, the peak’s center *a*_1_, the half width at half maximum *a*_2_, and the indicated shape *a*_3_ (0 for a purely Lorentzian and 1 for a purely Gaussian profile).

Due to the asymmetry of the G peak profile, a Pearson type-IV fit was applied, following the equation [35,37]:


[2]

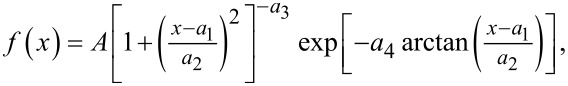



where *A* is a parameter that involves the amplitude parameter *I* and complex gamma and beta functions, and depends on the shape parameters *a*_2_, *a*_3_, and *a*_4_:


[3]

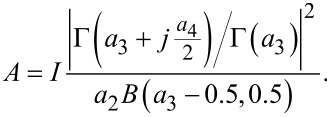



Here, *a*_1_ parameter refers to peak position, *a*_2_ refers to peak width, *a*_3_ indicates the “sharpness” of the peak, and *a*_4_ determines the peak’s asymmetry (tail or front). The fitting procedure involved the use of the SciPy Python package [38].

### FTIR

To investigate the oxygen functionalities within the rGO paper, Fourier-transform infrared (FTIR) spectroscopy was applied in attenuated total reflectance mode. The equipment involved a Perkin Elmer Frontier FTIR spectrometer. The spectra were obtained in the 520–4000 cm^−1^ wavenumber range with baseline calibration and normalized (0 to 100).

### XPS

X-ray photoelectron spectroscopy was applied to determine the surface concentrations of chemical bonds. The equipment applied was a PHI VersaProbeII Scanning XPS system with monochromatic Al Kα (1486.6 eV) X-rays (100 μm spot focused). High-energy-resolution spectra were obtained with 46.95 eV (0.1 eV step) pass energy in the analyzer and the photoelectron take-off angle at 45°. In order to maintain a constant sample surface potential, a dual beam charge compensation with 7 eV Ar^+^ ions and 1 eV electrons was used. The aliphatic carbon C 1s line at 285.0 eV was used as a charge reference in the spectra. The data analysis was conducted using PHI MultiPak software (v.9.9.3); the background was removed using the Shirley method. Due to the geometry of the spectrometer, the information depth of this analysis can be estimated at about 5 nm.

### XRD

X-ray diffraction was performed with PANalytical Empyrean diffractometer with a Cu Kα (1.540598 Å) X-ray source. Applied parameters were 45 kV and 40 A. Graphene paper samples were cut to fit the holders. The applied step angle was 0.026261°.

### Electrical properties characterization

The electrical properties (sheet resistance and conductivity) of the G-Flake^®^ reduced graphene oxide paper samples were determined using HMS Ecopia 5500 equipment in Van der Pauw configuration. For this method, samples were cut to a size of 1 cm × 1 cm. The measurements were conducted at room temperature. Values reported in this work are the average of ten measurements.

### Galvanostatic charge–discharge tests

The prototype cells (Swagelok-type with stainless steel current collectors) for the galvanostatic charge–discharge tests were prepared in an argon-filled glovebox (MBraun). The materials were tested in a two-electrode configuration in a full-cell setup for practical reasons [39]. When applying such a configuration, one needs to remember that during data analysis, especially regarding differential analysis, the observed features refer to phenomena on both electrodes [40]. Lithium metal (Sigma-Aldrich/Merck) was applied as the negative electrode, and 0.15 mL of 1 M LiPF_6_ solution in EC/DMC (Sigma-Aldrich/Merck) served as the electrolyte. A Whatman GF/A filter was used as a separator. The current rates were calculated based on the targeted current density and active mass material, in this case, the mass of the positive electrode (the investigated graphene paper sample). For this testing method, rGO paper sheets were cut into circles of 10 mm diameter with a stainless steel hole punch and weighed (the mass of cut samples ranged between ca. 0.0010 and 0.0018 g, with an areal mass loading from 1.3 to 2.3 mg/cm^2^ and a density of 0.56 to 2.57 g/cm^3^). Since the reduced graphene oxide paper sheets were free-standing, no additional current collector was applied for these tests.

The assembled prototype cells underwent galvanostatic charge–discharge tests (using Atlas 0961 Multichannel Battery tester) at room temperature. Starting after 20 h of rest after assembly, the cycling (between 0.01 and 3.20 V) involved steps of the following current rates: 5 × 10 mA/g, 5 × 30 mA/g, 5 × 50 mA/g, 5 × 100 mA/g, and 5 × 10 mA/g. Coulombic efficiency, defined as the ratio of discharge capacity to charge capacity, within each cycle was calculated [41].

## Results and Discussion

**Figure 2:**
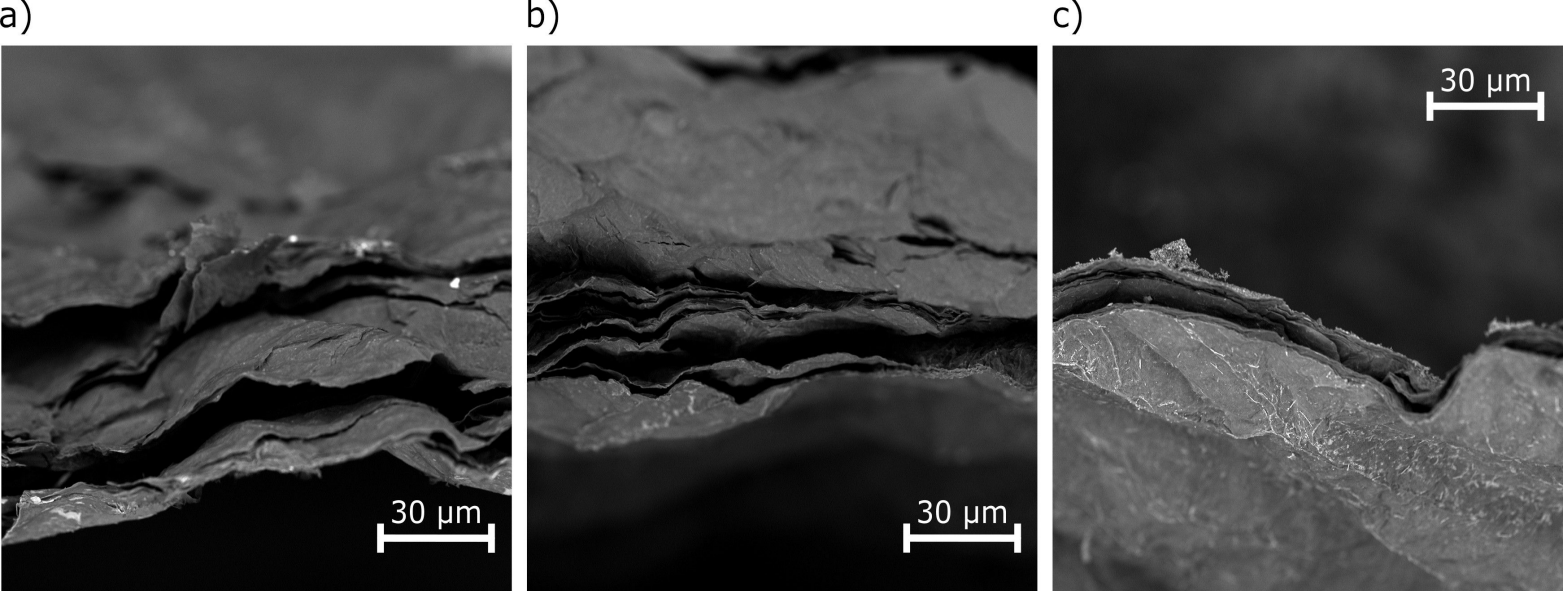
SEM images of the reduced graphene oxide paper cross sections: (a) T400, (b) T600, and (c) T800.

SEM images are presented in Figure 2a–c. With these, the rGO paper sheet thickness was determined (Table 1), reaching from 4.95 μm for the initial M300 material [34] up to 36.48 μm after the thermal treatment. The expansion of the fabricated films originates from thermal exfoliation [42]. The cross-sectional images of the samples revealed the presence of air pockets, another result of the release of gaseous products during reduction [31,43]. These features, along with the more effective removal of oxygen functionalities for sample T800, probably resulted in a lower increase in the final thickness of the sheet compared to T400 and T600, as the created air pockets collapsed. The morphological changes observed herein, likely resulting from the gaseous products of reduction and the intensity of their release, were similar to those reported by Kwon and colleagues [44]. Since the thicknesses of the presented rGO paper sheets were smaller than or close to 36 μm, it can be assumed that the release of gaseous products occurred through paths perpendicular to the surface of the rGO paper. Given the fact that no destruction of rGO sheets nor disruption of the film integrity was observed, we postulate that the gas release was rather a continuous phenomenon, driven by the heating process. The exposition of the flake edges and paths resulting from the removal of the gaseous products can be beneficial regarding the electrode design for cells operating liquid or gel electrolytes, ensuring improved transport and accessibility for lithium ions.

**Table 1:** Summarized electrical and chemical properties.

Sample	Thickness [μm]	O_2_ [%]	Sheet resistance [Ω/*□*]	Conductivity [S/cm]

M300	4.95 [34]	22.4 [34]	1088	1.86 [34]
T400	28.51	23.1	406	0.85
T600	36.48	17.0	102	2.72
T800	10.31	9.1	14	69.93

**Figure 3:**
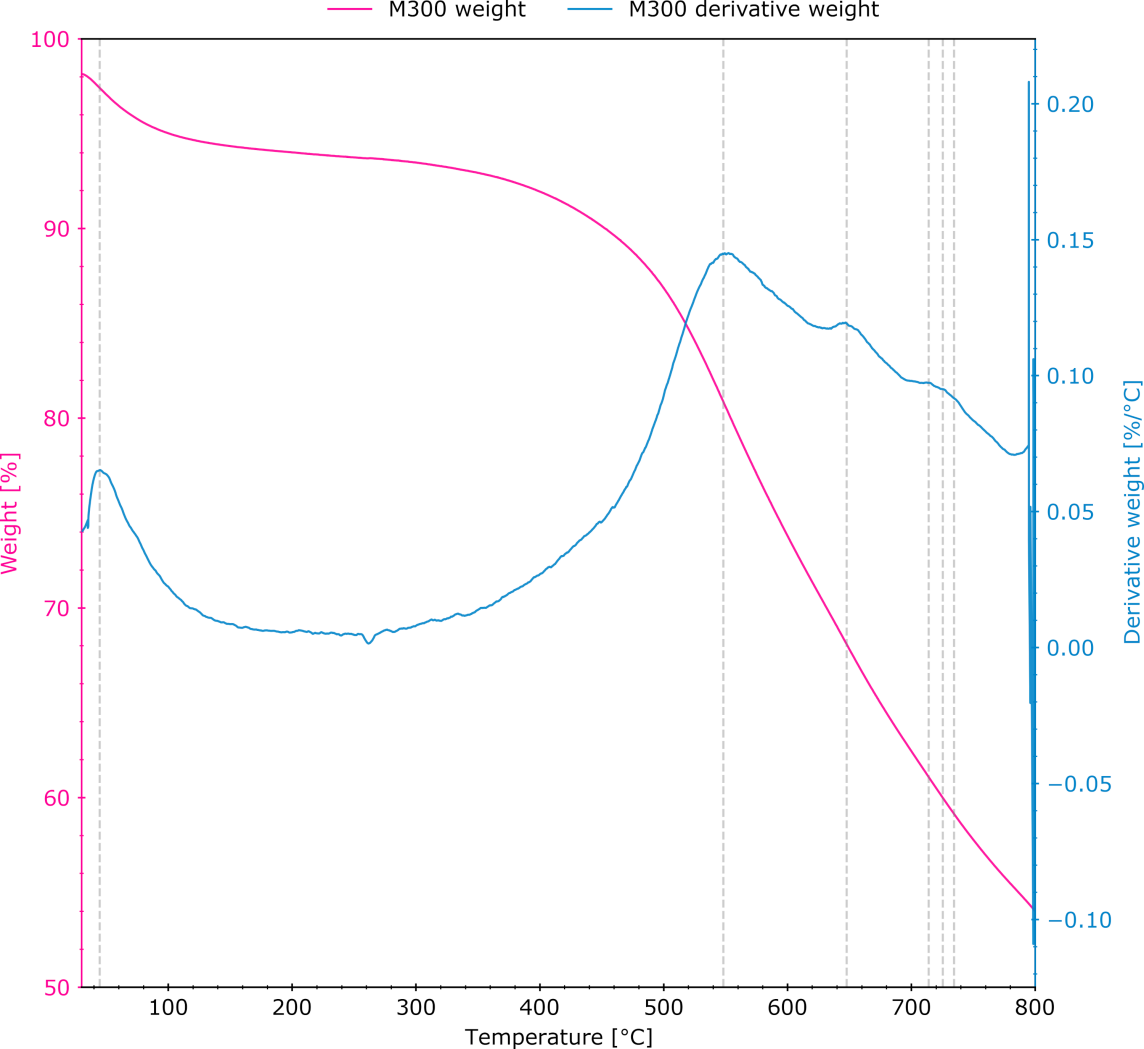
Thermogravimetric analysis (pink curve) and first derivative of the weight change (blue) of the M300 sample.

Thermogravimetric analysis (Figure 3) was performed on the M300 sample in order to determine the temperatures corresponding to the maximum rates of weight change resulting from the removal of oxygen functional groups. Differential analysis of the TGA curve revealed local maxima at 45, 548, 648, 714, 726, and 735 °C. The low-temperature weight loss contribution results from moisture removal. Since the initial material was previously mildly reduced at temperatures up to 300 °C, the weight loss in this range is moderate. The highest rates of reduction at 548 °C may be related to the loss of carboxyl, ether, and ketone groups [27].

The combustion elemental analysis, focused on the determination of the oxygen percentage (Table 1), revealed a value for the T400 sample (23.1% O_2_) that is close to that of M300 (22.4%), being within the range of measurement uncertainty (ca. 0.4%). As expected, the oxygen percentage for the materials annealed at higher temperatures decreased, reaching 17.0% and 9.1% for samples T600 and T800, respectively. As reported by Acik et al. [27], a 250–650 °C temperature range contributes to the removal of almost all oxygen functionality types, including C=O groups. Applying a temperature above this range allows ketone and ether functional groups to remain. Thus, to enable partial preservation of the ketone groups (considered as the redox-active sites), 800 °C was chosen as the reduction temperature.

With the lowest oxygen percentage and an increase in thickness, as well as the highest intensity of the Raman G peak, among the samples that underwent further thermal reduction, T800 was characterized by the highest conductivity of 69.93 S/cm (Table 1). The decrease in conductivity of the T400 sample (0.85 S/cm vs 1.86 S/cm for M300) results from a significant increase in its thickness. Successful thermal reduction was proved by the decrease in sheet resistance, starting from 1088 Ω/*□* for sample M300, through 406 and 102 Ω/*□* for samples T400 and T600, respectively, ending with 14 Ω/*□* in the case of T800.

**Figure 4:**
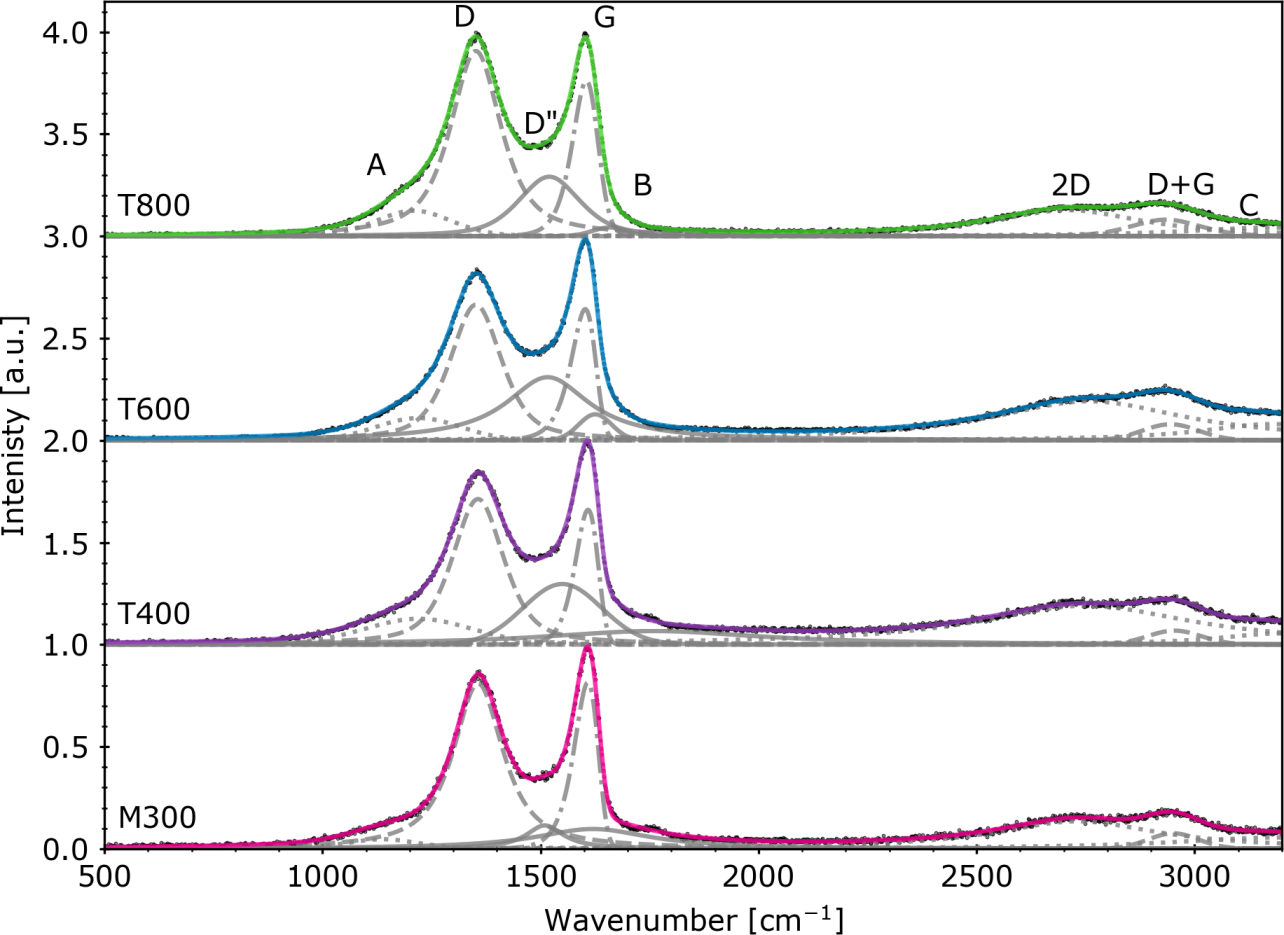
Raman spectra with the fitted functions (Table 2). The normalized data are marked with black points. Individual fitted peaks are represented with gray lines. Colored lines depict fitted functions (sum of peaks).

Raman spectra with fitted functions are presented in Figure 4. The A peak (also denoted as D^*^) possibly involves the C–H modes of the sp^2^ rings [21] or results from bonds between the sp^2^ and sp^3^ domains [36]. The C peak may also be of this origin [21]. The D peak refers to sp^2^ aromatic rings and their defects [21] and is also influenced by graphite oxidation [36]. The D'' peak is related to amorphous phases [36]. As described by Ferrari et al. [45], the G peak refers to sp^2^-bonded atoms present in both the rings and chains (functional groups). The 2D peak can be considered as representing the second order of this mode [36] or the D peak [45]. The peak denoted as B (also denoted as D') is said to be related to Stone–Wales defects (5-7-7-5 rings), 5-8-5 rings [21], and other irregularities in the carbon rings. The D+G peak is of graphene/graphitic origin [21].

**Table 2:** Intensities of the Raman peaks (fit procedure results).

Sample	*I*_A_ [a.u.]	*I*_D_ [a.u.]	*I*_D''_ [a.u.]	*I*_G_ [a.u.]	*I*_B_ [a.u.]	*I*_2D_ [a.u.]

M300	0.05	0.81	0.11	0.81	0.10	0.13
T400	0.13	0.71	0.30	0.66	0.07	0.19
T600	0.11	0.66	0.31	0.64	0.13	0.19
T800	0.12	0.91	0.29	0.76	0.04	0.13

Upon reduction, the A and D'' peaks increased, suggesting an increased number of sp^3^-hybridized carbon atoms and amorphous areas bonded to sp^2^-hybridized carbon atoms [36]. A significant increase of the D peak in the case of the T800 sample needs to be considered regarding the oxygen percentage change; the removal of such a large number of functional groups resulted in defect formation within the graphene structure. However, the G peak intensity behavior after thermal annealing at various temperatures suggests that higher temperatures promote better preservation of areas with sp^2^-bonded atoms. This peak can also involve a contribution from sp^2^-hybridized carbon atoms in carbonyl groups (possibly ketones). The intensities of deconvoluted peaks were summarized in Table 2.

**Figure 5:**
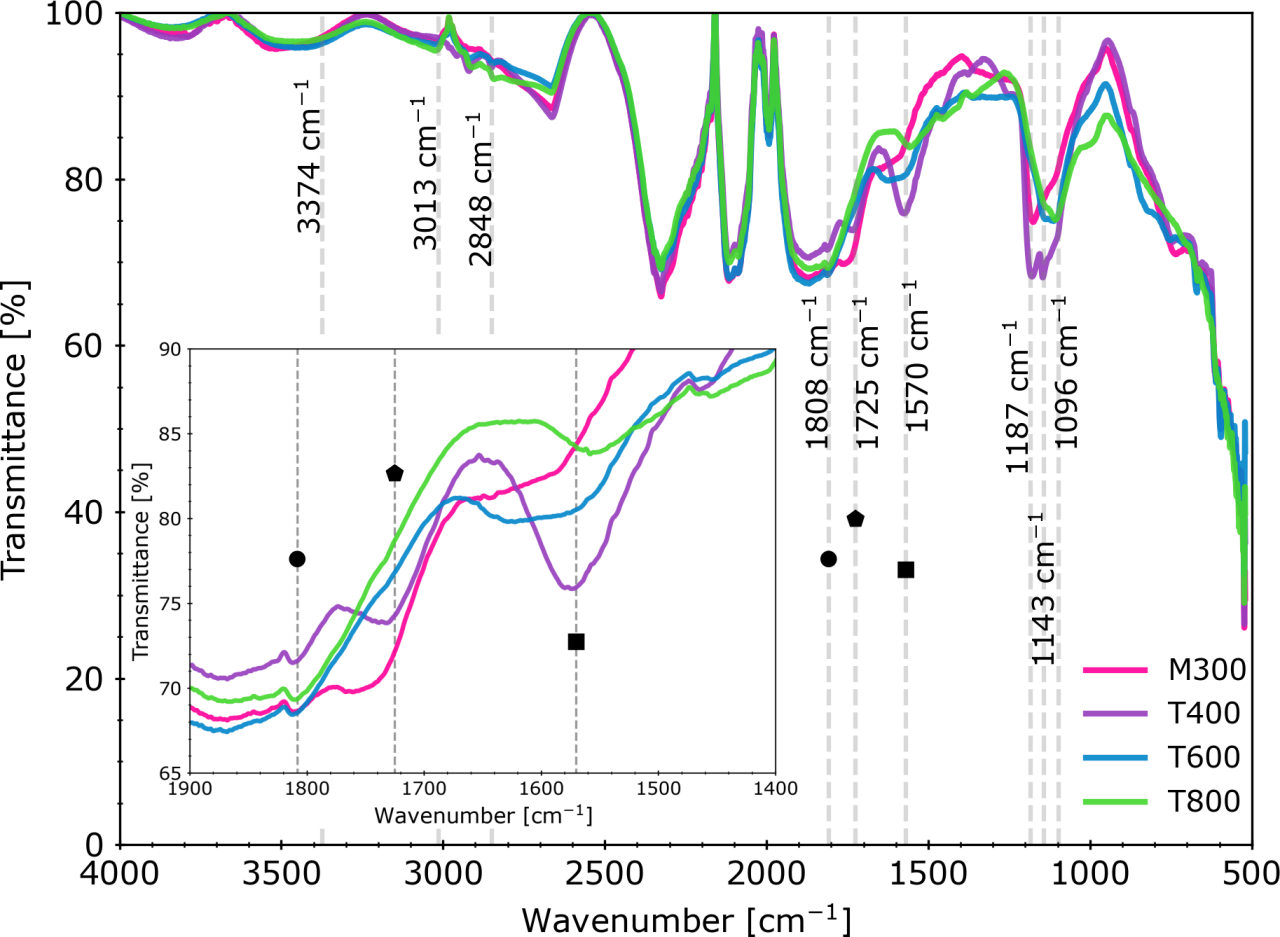
FTIR spectra of the thermally reduced graphene oxide paper samples. The inset graph shows the wavenumber range characteristic for C=O-related vibrations. Bands near 1570, 1725 and 1808 cm^−1^ are marked with a square, a pentagon and a circle, respectively.

The transmittance spectra of the prepared rGO paper sheets (Figure 5) revealed bands at ≈1096, ≈1143, and ≈1187 cm^−1^, which could be assigned to aliphatic ketones (“C–C(=O)–C bending in the C–C–C group”) [46]; however, they can also origin from –OH groups as well as ether (C–O) and epoxide (C–O–C) groups [27,46]. The band near ≈1570 cm^−1^ can be interpreted as a result of stretching vibrations of the C=C bonds in the aromatic lattice [27,46]. Another origin of this band might be the bending vibrations in water molecules [46]. The distinct peaks observed between 1700 and 1800 cm^−1^ correspond to the C=O stretching vibrations from carbonyl groups [27].

The peak near 2848 cm^−1^ can refer to the C–H stretching in aldehyde groups [46] and the 3013 cm^−1^ peak to the aromatics C(sp^2^)–H [46]. The wavenumber shift in relation to literature data can result from the applied reduction parameters (cf. Xiong et al. [47]). The band observed in the wavenumber range of 3300–3600 cm^−1^ can be attributed to the O–H stretching vibrations from hydroxy groups or adsorbed water. The temperature values for samples T600 and T800 changed the FTIR spectra significantly compared to sample T400. Samples T600 and T800 exhibited also weaker peak signal for 1187, 1570, and 1725 cm^−1^, possibly due to COOH functional group loss [27] and partial ketone removal. The broad band located at ≈1808 cm^−1^ remained. What is also worth mentioning is that the peak at ≈3013 cm^−1^ was more pronounced in all samples except T400.

**Figure 6:**
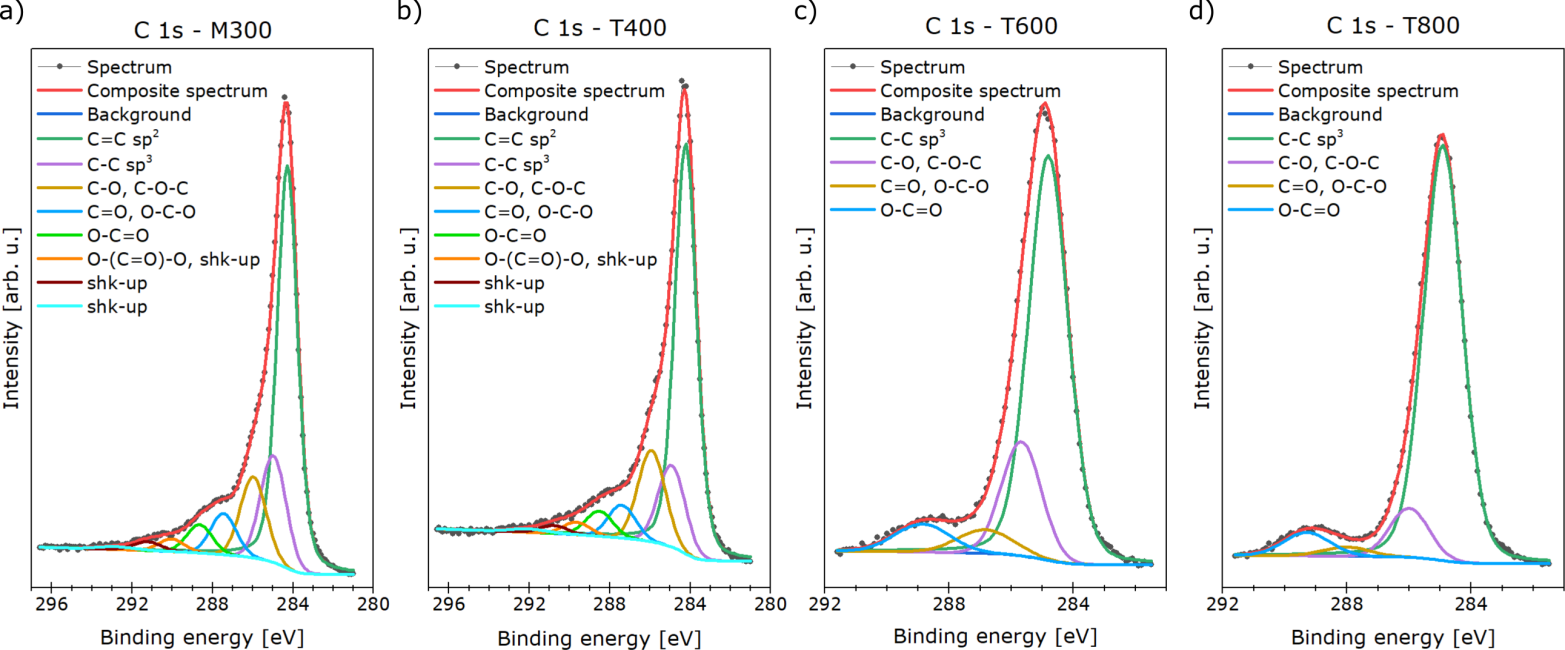
Deconvolution of the carbon-related region in the XPS spectra of the samples (a) M300, (b) T400, (c) T600, and (d) T800.

The results of XPS spectra deconvolution are summarized in Table 3 and Table 4. The carbon-related region (depicted in Figure 6) revealed sp^2^ bonds for samples M300 and T400 [48]. In these materials, also shake-up-related excitations were identified [49]. Both features confirm aromatic structures within the investigated samples [48,50]. However, they were not identified in samples T600 and T800, probably due to highly defected surface of these materials (taking into consideration the 5 nm information depth of XPS). It is worth noting that the samples T600 and T800 contain ca. 22% ketone-related bonds, which predispose them for electrochemical applications.

**Table 3:** Surface composition (atom %) determined by fitting XPS spectra: samples M300 and T400.

	C								O		Si	S	Ca

binding energy [eV]	284.3	285.0	286.0	287.5	288.6	289.6	290.7	292.7	531.0	532.5	102.7	168.7	347.7
groups/oxidation state	C=C sp^2^	C–C sp^3^	C–O–C; C–OH	C=O; O–C–O	O–C=O	O(C=O)O shk-up	shk-up	shk-up	O=C O–S	O–C O–Si	silicates	SO_4_^2−^	Ca^2+^
M300	46.3	13.3	10.1	5.1	3.5	1.6	1.2	0.5	6.9	10.0	0.3	0.8	0.3
T400	33.8	7.4	8.3	3.0	2.3	1.1	0.7	0.4	1.9	31.7	6.3	1.8	1.5

**Table 4:** Surface composition (atom %) determined by fitting XPS spectra: samples T600 and T800.

	C				O			Na	Mg	Si	S	Ca	Mn

binding energy [eV]	285.0	286.0	287.8	289.2	530.0	531.9	533.3	1072.0	1303.8	102.7	168.7	347.7	640.8
groups/oxidation state	C–C	C–O C–O–C	C=O O–C–O	O–C=O	O–Mn	O–Si O=C O–S	O–C –OH	Na^+^	Mg^2+^	silicates	SO_4_^2−^	Ca^2+^	Mn^3+^
T600	41.9	10.9	3.1	3.7	6.5	19.0	3.5	0.2	0.8	0.5	3.3	4.7	2.1
T800	50.8	5.5	1.2	3.1	5.6	20.7	1.4	1.6	1.0	0.7	2.3	5.1	1.1

**Figure 7:**
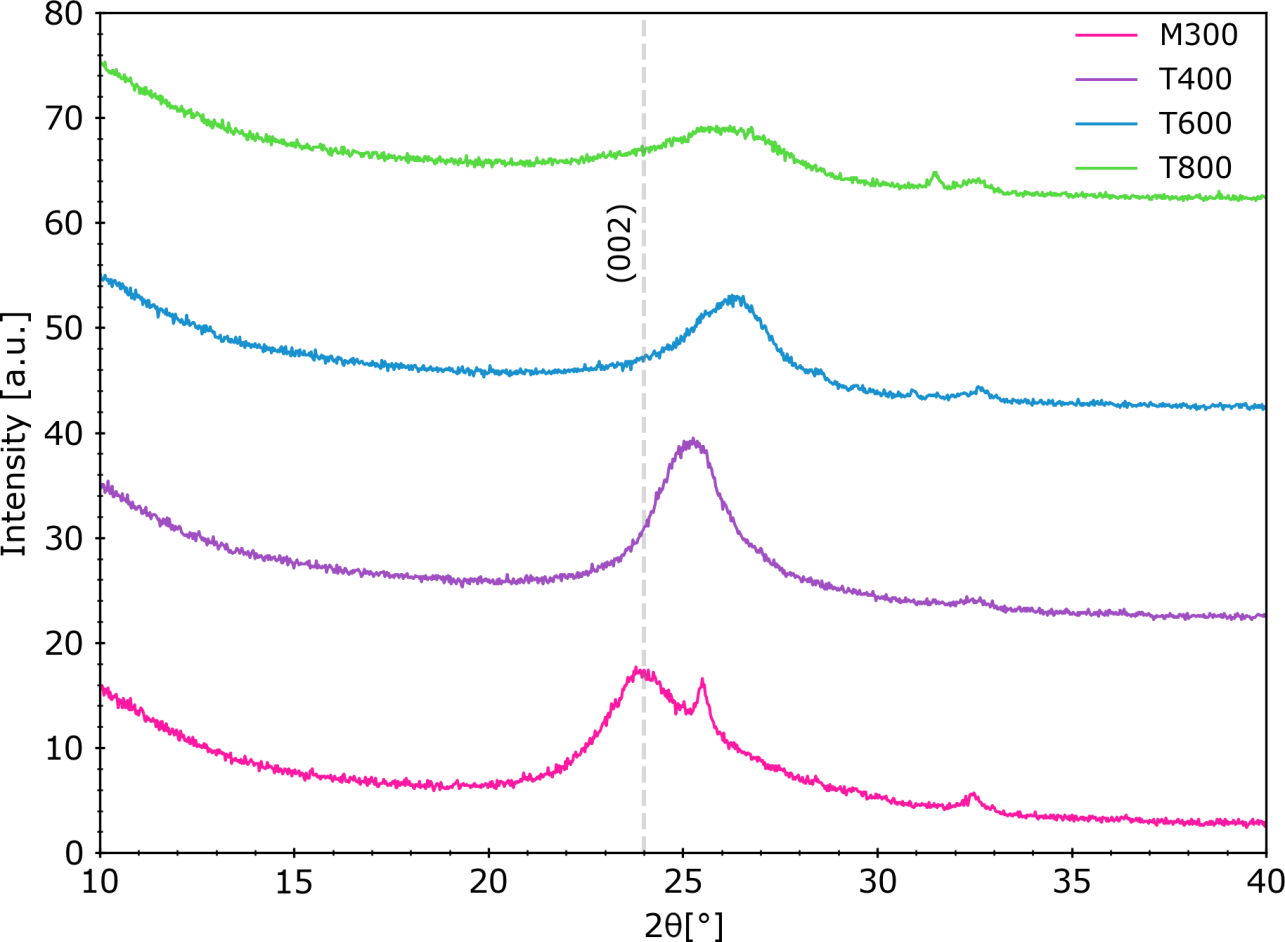
Diffractograms of rGO paper samples. Visible changes in the (002) peak shape confirm partial restoration of the graphitic structure.

The XRD results of rGO paper sheets are depicted in Figure 7. With increasing reduction temperature, the peak of the (002) plane, which corresponds to an ordered crystalline structure typical of graphite, located around 2θ ≈ 24°, becomes broader and less intense [51]. These changes confirm the progressive reduction of graphene oxide, associated with a partial restoration of the graphitic structure. The slight shift in peak position suggests that the interlayer spacing remains relatively stable, while higher temperatures, particularly 800 °C, may lead to partial degradation and increased structural disorder.

**Figure 8:**
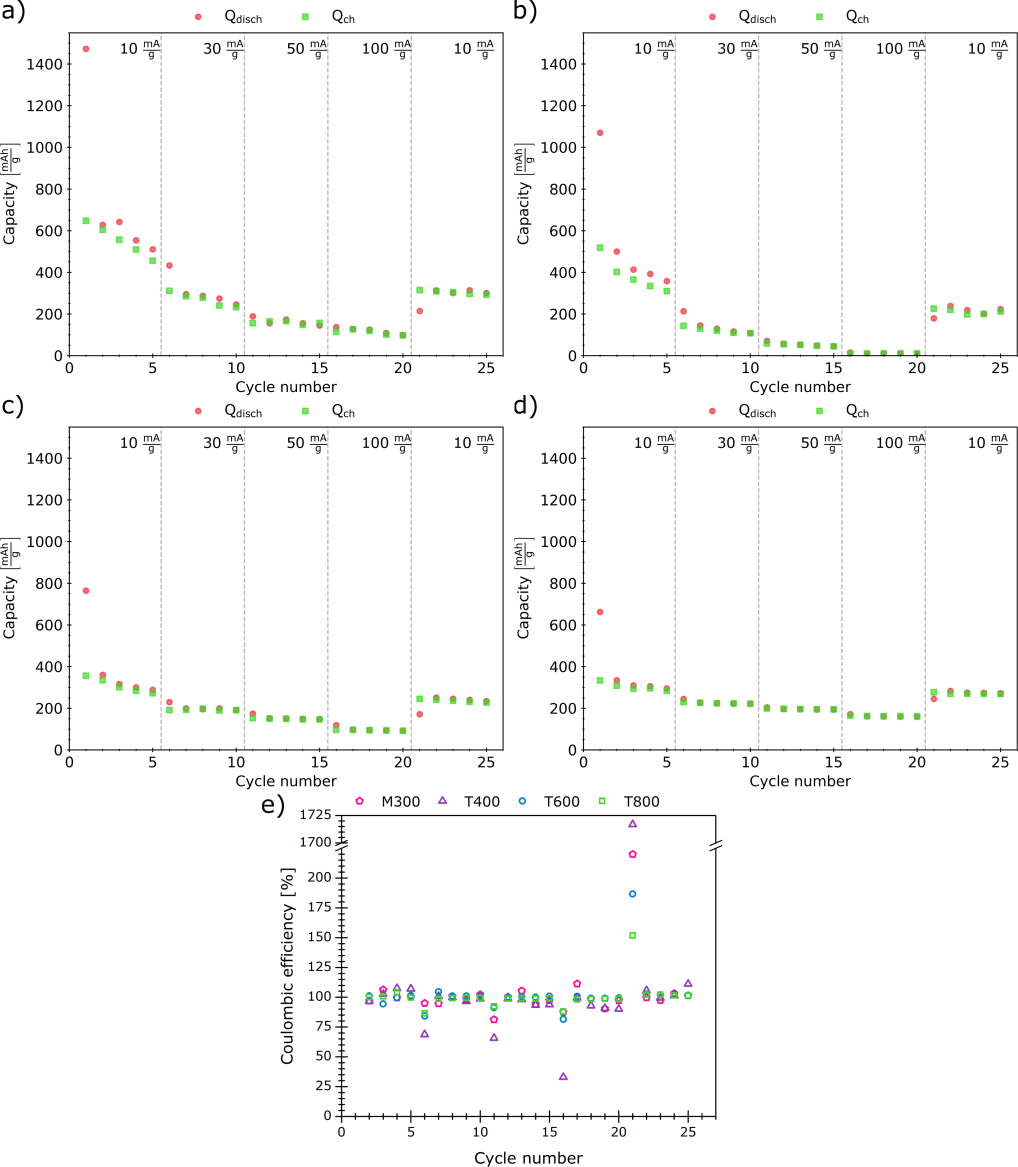
Results of the galvanostatic charge (green)–discharge (red) tests. The capacity is shown as a function of the cycle number for samples (a) M300, (b) T400, (c) T600, and (d) T800. Coulombic efficiency for each sample is also plotted (e).

The obtained rGO paper sheets underwent electrochemical properties characterization; galvanostatic charge–discharge tests were performed for prototype cells with rGO paper samples as cathode material (Figure 8). In all samples, one can notice capacity fading within the first five cycles (at 10 mA/g current density) with a significant drop in capacity after the first discharge of the cells, reaching 510, 358, 288, and 295 mAh/g for discharge and 456, 310, 272, and 284 mAh/g for charge for samples M300, T400, T600, and T800, respectively, in the fifth charge–discharge cycle. Such a behavior stems from the formation and growth of a solid electrolyte interface on the negative electrode [52].

The current density increase during these tests led to further loss in capacity. For a current density of 30 mA/g, the discharge capacity values dropped to 246, 109, 190, and 221 mAh/g for samples M300, T400, T600, and T800; the discharge capacity dropped further to 145, 45, 148, and 193 mAh/g when a current density of 50 mA/g was applied. At 100 mA/g, samples T400 and T600 revealed capacity values below 100 mAh/g. The samples M300 and T800 also exhibited capacity loss with increasing current densities. However, sample T800 was more stable upon cycling with capacity values of 160 mAh/g for 100 mA/g current density. During the last cycles (with the current density set back to 10 mA/g), the discharge capacities reached 300, 223, 235, and 272 mAh/g for samples M300, T400, T600, and T800, respectively.

The Coulombic efficiency plots in Figure 8e prove that a higher thermal reduction temperature results in a reduced spread of these parameters’ values. It is also worth mentioning that the coulombic efficiency for the T800 sample demonstrated lower variability after current density changes.

**Figure 9:**
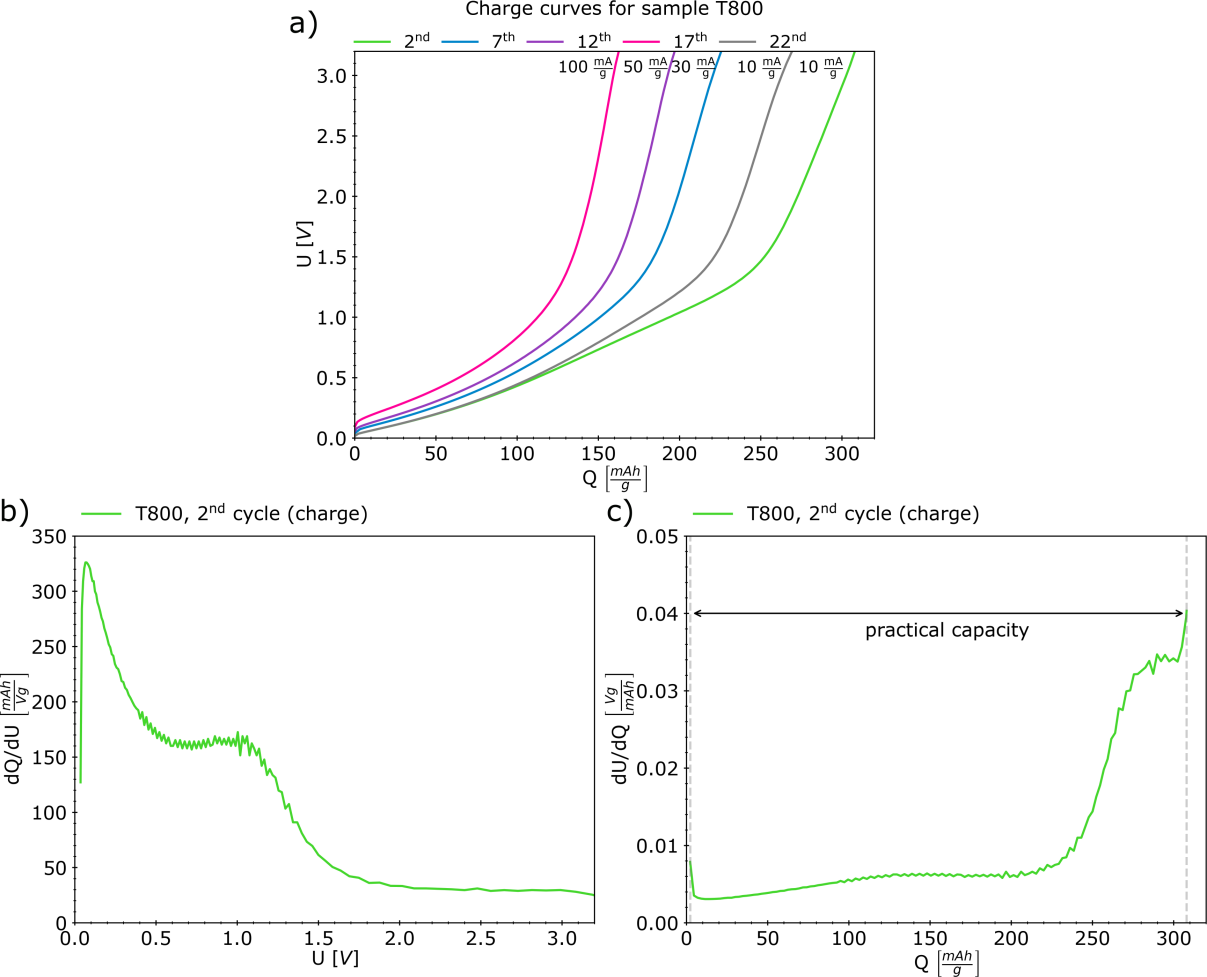
(a) Charge curves for sample T800 and (b, c) differential analysis: (b) differential capacity and (c) differential voltage.

The galvanostatic charge–discharge test results (i.e., charge curves and differential analysis) of sample T800 are presented in Figure 9. One needs to remember that the applied full-cell testing method reveals features appearing concurrently on both electrodes [40] (in our case, Li metal anode and rGO paper cathode). Peaks in differential capacity plots indicate lithiation equilibria [40]. The broadening of peaks (centered at ≈0.07 and ≈1.0 V) in the differential capacity plot may result from defects in the material structure [53]. These peaks correspond to plateau regions in the differential voltage plots, whereas peaks in the d*U*/d*Q*(*Q*) plot, refer to transitions between these equilibria [40]. For the T800 sample, a broad peak was observed centered at ≈250 mAh/g. This plot type allows one to predict the practical capacity of the cell, which is considered the width of the well created by the curve [40]; here, the full practical capacity of the cell determined for the second charge reached ≈306 mAh/g. As described by Yadav et al. [53], the charge storage mechanism in rGO materials cannot be described as a typical “staging mechanism”.

## Conclusion

In this paper, the authors presented preliminary data on variants of the thermal reduction of reduced graphene oxide paper for an optimized synthesis as an electrode material. The applied methods revealed the influence of thermal treatment parameters on the chemical and physical properties of the obtained materials and, as a result, on their electrochemical performance characteristics. In particular, upon raising the reduction temperature, the character of the functional groups changed, as their partial elimination occurred with the preservation of the ketone-related band near ≈1800 cm^−1^. Thus, the charge storage mechanism via redox reactions involving these functionalities was enabled, promoting the application of the material as lithium- or sodium-ion energy storage material [54]. Moreover, the stability of the cell’s performance was improved with increasing reduction temperature. Changes in structural properties revealed by Raman spectroscopy influenced the electrochemical properties of these materials, probably due to the decreased charge transfer resistance and balanced electronic and ionic conductivity [55]. Similar to the case reported herein, He et al. reported that higher intensity ratios of *I*_D_/*I*_G_ and *I*_D+D'_/*I*_2D_ (or *I*_D+G_/*I*_2D_, as denoted herein) were related to better electrochemical performance of the examined materials. Moreover, such an optimal additive improved the diffusion coefficient in a composite lithium cobalt oxide electrode [55]. Furthermore, the introduction of defects as a result of iodine doping reported by Li et al. resulted in improved sodium ion storage and electron transport [56]. The carbon lattice in rGO flakes provides electrical conductivity, while defects and vacancies enable adsorption sites for electrolyte ions [57]. This research contributes to the development of thermal reduction methods of free-standing rGO thin films, that is, rGO paper. It is worth emphasizing that the presented material was prepared without any additional surfactants or binding agents; the reduced graphene oxide flakes are the sole component of the obtained films. Further research will include rGO paper functionalization to stabilize the material’s performance and improve its capacity.

The discharge capacity values for sample T800 reached at least ≈159 mAh/g at 100 mA/g current density. In comparison, the literature reported data for similar electrode materials made of rGO paper, such as research conducted by Ha et al. [7], proved stabilized capacities of 110 to 115 mAh/g when cycled with 137 mA/g current density; however, the mentioned research reported these values for a material reduced at 650 °C in a reducing atmosphere; also, a surfactant agent was applied for the preparation of the film. The capacity values presented herein are also close to those reported by Wen et al. for a non-nitrogen-doped graphene paper sample obtained via a hydrothermal process [58], with a gravimetric capacity below 200 mAh/g obtained during cycling at 100 mA/g current density.

The basic rGO paper parameters investigated herein allow for further development of graphene paper electrode materials with a particular focus on functionalization and the graphene paper thickness.

## Data Availability

Data generated and analyzed during this study is available from the corresponding author upon reasonable request.
